# Improvement of Mechanical Properties and Self-Healing Efficiency by Ex-Situ Incorporation of TiO_2_ Nanoparticles to a Waterborne Poly(Urethane-Urea)

**DOI:** 10.3390/polym11071209

**Published:** 2019-07-19

**Authors:** Iñigo Díez-García, Arantxa Eceiza, Agnieszka Tercjak

**Affiliations:** Group ‘Materials + Technologies’ (GMT), Department of Chemical and Environmental Engineering, Faculty of Engineering of Gipuzkoa, University of the Basque Country (UPV/EHU), Pza Europa 1, 20018 Donostia-San Sebastian, Gipuzkoa, Spain

**Keywords:** waterborne polyurethanes, titanium dioxide nanoparticles, nanocomposites, self-healing, conductive properties, mechanical properties

## Abstract

This research work was focused on the incorporation of TiO_2_ nanoparticles into synthesized solvent-free waterborne poly(urethane-urea) (WPUU) based on hydrophilic poly(ethylene oxide) (PU0) in order to improve both the mechanical properties and self-healing effectiveness of a polymer matrix. The incorporation of TiO_2_ nanoparticles resulted in a successful enhancement of the mechanical properties of nanocomposite films when compared to PU0. Simultaneously, the obtained nanocomposite films did not only maintain the self-healing ability of the PU0 film, measured by means of mechanical properties after successive cutting/recovery cycles, but they also showed a higher self-healing efficiency than the PU0 film. Moreover, the well-dispersed TiO_2_ nanoparticles, visualized by atomic force microscopy (AFM), kept their conductive properties when embedded in the PU0 matrix, as was confirmed by electrostatic force microscopy (EFM). This research work described a simple and industrially appealing way to control the dispersion of commercially available TiO_2_ nanoparticles in waterborne poly(urethane-urea) for the designing of inorganic/organic hybrid nanocomposites with enhanced mechanical properties and self-healing efficiency, in which TiO_2_ nanoparticles preserved their conductive properties within the polymer matrix.

## 1. Introduction

Nanocomposites (NC), consisting of a nanometric size inorganic phase distributed in a polymer matrix [1], have attracted a great deal of attention by virtue of their multiple fields of application. They can be used in a broad range of applications such as biomedicine, solar cells, sensors, batteries, photocatalysis, display panels, gas separation, water purification, and transistors [1,2,3].

In the case of waterborne polyurethanes (WPU), the addition of inorganic nanoparticles can enhance the properties of these polymers [4,5,6]. This is the consequence of the combination of the properties of the inorganic nanoparticles with the properties of the polymers [4,7] leading to better thermal [4,7] and mechanical properties [4,8,9]. Different types of inorganic nanosized fillers have been incorporated into WPU, such as Au [10], Ag [6,11], grapheme [11,12], different kinds of silica clays [7,13,14], CaCO_3_ [15], ZnO [5], Fe_3_O_4_ [16], and TiO_2_ [8,9,17]. Titanium dioxide (TiO_2_) is one of the most interesting inorganic nanofillers given its environmentally friendly nature, being chemically inert and showing both high photostability and photocatalytic activity [9,17]. On behalf of its photocatalysis, which can be used for the photodegradation of organic compounds [17], TiO_2_ displays self-cleaning [17,18] and also antibacterial activity [17], which are appealing for a potential industrial application in paints and coatings. Furthermore, TiO_2_ improves the UV resistance [19] and mechanical properties [20] of the polymer matrix. Additionally, due to the n-type semiconducting nature of TiO_2_ [4,21], it can even confer conductivity [3] to nanocomposites. 

In order to obtain satisfactory nanocomposites, a proper dispersion of the nanoparticles in the polymer matrix must be achieved. Nonetheless, nanoparticles tend to aggregate, forming clusters [15,17]. This happens due to their large surface area/particle size ratio [15,17], high surface tension [15], and/or incompatibility with the organic polymer matrix [15]. This highlights the importance of the approach taken for the dispersion of the nanoparticles. The sol-gel process [4,7] and physical mixing by ultrasonication [17] are widely used methods to obtain good dispersion. Nevertheless, from an industrial point of view, mechanical stirring would be more suitable. This approach has also been employed for TiO_2_ nanoparticle dispersion [8,17], though to an inferior extent. Nonetheless, usually, a better dispersion can be achieved by in situ incorporation during polymer synthesis [8,17].

WPU, like other polymers and materials, may suffer physical damage leading to scratches, microcracks, and larger cracks [22]. This damage affects their lifetime, which could, however, be extended if the WPU had the ability to repair themselves. Self-healing is the ability of materials to partially or completely repair themselves, recovering from the deterioration of their properties caused by the damage suffered [23]. There are two main approaches to self-healing: extrinsic and intrinsic. The first one consists of intentionally adding a healing agent that is generally microencapsulated [24,25,26,27]. The second, intrinsic self-healing, is achieved thanks to both the physical interaction between the damaged interfaces [26] and to the autonomous reversibility of covalent and non-covalent bonding in the polymer matrix [23,24,25,26]. These reversible interactions can occur due to the Diels–Alder reaction [28], disulfide bonds [22], alkoxyamines moieties [25], van der Waals [29], π-π stacking [30], ionic [26], and hydrogen bonding interactions [26,29,30], among others.

Since the inception of self-healing concepts [31,32], considerable research attention has been dedicated to this topic, given the benefits of extending the service life of materials. In the case of polyurethanes, some research has been done on their self-healing properties using different approaches [25,33,34]. However, there is still not much research being conducted on the self-healing properties of WPU [22,26,35]. Taking into consideration the hydrogen bonding between urethane groups [24,26], polyurethanes are of great interest for the development of novel self-healable polymers. Furthermore, poly(urethane-urea) is even more appealing due to the presence of urea groups, which provide reinforcement concerning hydrogen bonding sites [26], as there is one more N–H group than in urethane. 

In our previous work, the synthesis of a waterborne poly(urethane-urea) (WPUU) dispersion based on poly(ethylene oxide) (PEO) as a macrodiol was reported [36]. The aim of the present work was the fabrication of nanocomposites using a commercial aqueous solution of TiO_2_ nanoparticles by their ex situ dispersion in WPUU containing hydrophilic PEO segments, which could interact with the TiO_2_ nanoparticles [3], modulating the self-healing properties of the matrix. This WPUU contained sulfonate groups, which can play the role of an ionic internal emulsifier. Additionally, these ionic groups may contribute to the self-healing process as a reversible interaction [26], together with the previously mentioned hydrogen bonding. In addition to all of the above, this work also focused on studying the self-healing ability of the prepared WPUU and NC films.

In the present work, the chemical structures of WPUU and NC films were studied by Fourier transform infrared spectroscopy (FTIR). The thermal behavior was analyzed using differential scanning calorimetry (DSC) while thermal stability was determined by thermogravimetric analysis (TGA). The morphology of the nanocomposite films was studied by atomic force microscopy (AFM) and optical microscopy (OM). The self-healing ability was investigated by means of mechanical properties using an Instron tensile testing machine. Furthermore, the conductive properties of the TiO_2_ nanoparticles embedded in the polymer matrix were studied by means of electrostatic force microscopy (EFM).

## 2. Experimental Methods

### 2.1. Materials

The employed diisocyanate was diphenylmethane diisocyanate (MDI), kindly provided by Covestro (Darmstadt, Germany). A chain extender and internal emulsifier, 2,4-diamino-benzenesulfonic acid (DBSA), supplied by Sigma Aldrich (Japan) and neutralized with NaOH, was used [37]. The soft segment of the synthesized waterborne poly(urethane-urea) dispersion was formed by poly(ethylene oxide) (PEO) with a weight average molecular weight, *M*_w_*,* of 1000 g mol^−1^, as indicated by Sigma Aldrich (St. Louis, MO, USA). In addition, the mixture of rutile and anatase TiO_2_ nanoparticles (particle size <150 nm) were also supplied by Sigma-Aldrich (Darmstadt, Switzerland). Table S1 in Supplementary Materials displays more details regarding the reagents.

### 2.2. Synthesis of the Waterborne Poly(Urethane-Urea)

Waterborne poly(urethane-urea) dispersion was synthesized as previously reported by us [36]. Briefly, the MDI and PEO were left to react for 2 h at 80 °C before cooling down to room temperature and adding the neutralized chain extender DBSA to the water. Afterwards, it was supplemented with more water for proper dispersion. Table 1 displays information regarding the synthesized PU0 also reported in reference [36].

### 2.3. Preparation of Nanocomposites Films

**Figure 1:**
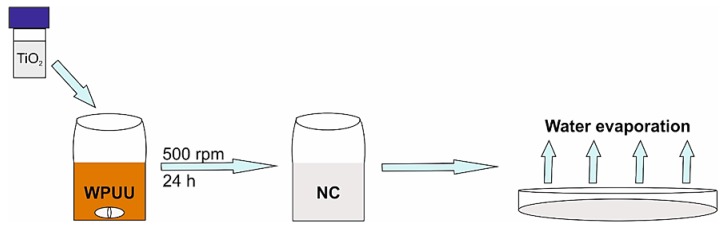
Schematic description of the procedure for the preparation of the nanocomposites (NCs). WPUU = waterborne poly(urethane-urea).

Once the WPUU dispersion was synthesized, the solid content was calculated in order to prepare nanocomposites by ex situ physical mixing of commercial aqueous solution of TiO_2_ nanoparticles in a WPUU dispersion (Figure 1). The density and solid content of the commercial aqueous solution of TiO_2_ nanoparticles were taken into account for the preparation of the nanocomposites. Three different TiO_2_ weight content nanocomposites (10, 20 and 40 wt %) were prepared regarding the solid content of WPUU dispersion and the TiO_2_ aqueous solution. In addition, a WPUU film without TiO_2_ was also prepared as a reference. The samples were denoted as PU0, for the matrix without inorganic nanoparticles, and XTiO_2_-PU0 for the nanocomposites, where X can be correlated to the TiO_2_ wt % content.

For the physical mixing of the PU0 and TiO_2_ nanoparticles in the commercial aqueous solution a magnetic stir bar was used to keep the mixtures stirring for 24 h at 500 rpm (Figure 1). White homogeneous dispersions were obtained.

For the preparation of the nanocomposite films, 8 mL of each mixture was poured onto glass surfaces covered with Teflon (Figure 1). In order to remove any air bubbles that could disrupt the surface of the films, they were kept for 5 days in a vacuum. After this, they were left drying at room temperature for one day, and then kept in a vacuum at 60 °C to prevent water absorption by the investigated PU0 and NC films. During the entire preparation process, both mixtures and NC films were protected from exposure to light.

### 2.4. Characterization Techniques

#### 2.4.1. Solid Content

Solid content was calculated by drying a volume of PU0 in an oven at 105 °C for 3 h. It was calculated in the same way as reported before by us [36].

#### 2.4.2. Fourier Transform Infrared Spectroscopy

A Nicolet Nexus 670 spectrometer (Madison, WI, USA) provided with a MKII Golden Gate accessory (Specac) with a diamond crystal at a nominal incidence angle of 45° and ZnSe lens was used to characterized the functional groups of PU0 and TiO_2_-PU0 nanocomposite films by Fourier transform infrared spectroscopy (FTIR). The range was between 4000 and 650 cm^−1^, with a resolution of 8 cm^−1^ and averaging 32 scans.

#### 2.4.3. Differential Scanning Calorimetry

To determine the thermal properties of PU0 and TiO_2_-PU0 nanocomposite films, a DSC 3+ Mettler Toledo with an autosampler and an electric intracooler as refrigerator unit was employed (Columbus, OH, USA). Differential scanning calorimetry (DSC) scans were carried out from −90 °C to 200 °C by heating the weighted sample (between 5 and 8 mg) encapsulated in an aluminum pan at a scanning rate of 5 °C min^−1^ in an inert nitrogen atmosphere.

#### 2.4.4. Thermogravimetric Analysis

The thermal stability of PU0 and TiO_2_-PU0 nanocomposite films was determined by thermogravimetric analysis (TGA) using a TGA/SDTA 851 Mettler Toledo (Columbus, OH, USA). Between 5 mg and 10 mg of each sample were exposed to a dynamic run from 25 to 800 °C, at a heating rate of 10 °C min^−1^ in nitrogen atmosphere.

#### 2.4.5. Atomic Force Microscopy

Atomic force microscopy (AFM) captured images of the morphology of PU0 and TiO_2_-PU0 nanocomposite films at room temperature by applying a resonance of 320 kHz, collecting the height and phase images simultaneously. The obtained AFM images were similar and, for this reason, only phase images are shown. A Nanoscope V scanning probe microscope (Multimode 8 Bruker Digital instruments) with an integrated force generated by cantilever/silicon probes with a tip radius of 5–10 nm and a length of 125 µm was utilized (Billerica, MA, USA). Different areas of the films were scanned to ensure that the obtained morphology was representative and homogeneous at a microscopic level.

#### 2.4.6. Optical Microscopy

In order to ensure that PU0 and TiO_2_-PU0 nanocomposite films were homogeneous at a microscopic level, optical microscopy (OM) was employed. Images were taken with the ×10 objective of a Nikon Eclipse E600 (Melville, NY, USA).

#### 2.4.7. Mechanical Testing/Self-Healing Ability

Mechanical properties and the self-healing ability of PU0 and TiO_2_-PU0 nanocomposite films at room temperature were studied by tensile test using an Instron 5967 testing machine provided with a 500 N load cell and pneumatic grips to hold samples (Norwood, MA, USA). At least 6 specimens (10 mm × 4.5 mm × 0.3 mm) of each composition were tested (original specimens and healed specimens). All investigated films were kept at 60 °C in a vacuum before testing was carried out at a crosshead speed of 10 mm min^−1^. For the self-healing measurement, specimens were cut and put into contact for 23 h in a desiccator and then kept for 1 h at 60 °C in a vacuum before testing. The time between the specimen cut and testing after healing was established to be 24 h. Tensile modulus (*E*), tensile strength (*σ_max_*), stress at break (*σ_b_*) and elongation at break (*ε_b_*) were determined from the obtained stress–strain curves.

The ratio of elongation at break, tensile strength, stress at break and modulus of the healed specimen to that of the original specimen were taken to determine the healing efficiency.

(1) $$Healing\;efficiency\;=\;\frac{Mechanical\;Property\;\left(healed\right)}{Mechanical\;Property\;\left(original\right)}\times 100$$

#### 2.4.8. Contact Angle/Self-Healing

The hydrophilic nature of PU0 and TiO_2_-PU0 nanocomposite films was analyzed by static water contact angle (WCA) using an SEO Phoenix300 (SEO, Suwon, Korea) at room temperature. At least 10 measurements were performed by sessile drop method for each film. A deionized water droplet was deposited on the surface of the film by syringe tip. The Young–Dupré equation (Equation (2)), where *W_a_* corresponds to the work of adhesion (mN m^−1^), *γ* to the surface energy (mN m^−1^) and *σ* to the contact angle (radians), allowed us to calculate the surface energy of each film [38], both original and self-healed.

(2) $$W_{a}=\;\gamma (1+cos\sigma )$$

#### 2.4.9. Electrostatic Force Microscopy

The conductive properties of TiO_2_-PU0 nanocomposite films were studied by electrostatic force microscopy (EFM). EFM measurements were performed using the Dimension Icon scanning probe operating in lift mode (lift height: approximately 400 nm) equipped with a Pt/Ir coating tip with a resonance frequency around 75 kHz (Billerica, MA, USA). A voltage was applied to the cantilever tip in order to detect the secondary imaging mode derived from the tapping mode that measures the electric field gradient distribution. This technique allows us to measure the qualitatively conductive properties at the nanometric level of TiO_2_ nanoparticles embedded in the PU0 matrix.

## 3. Results and Discussion

### 3.1. FTIR Spectra

**Figure 2:**
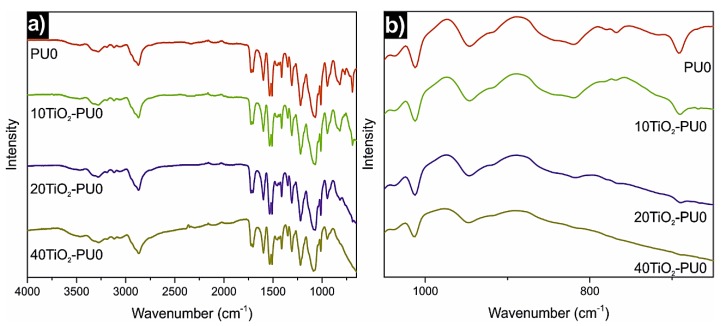
FTIR spectra of (**a**) the PU0 and TiO_2_-PU0 nanocomposite films and (**b**) amplification of the area around 800 cm^−1^.

Figure 2 displays the FTIR spectra of PU0 and TiO_2_-PU0 nanocomposite films. As shown, the spectra of both PU0 and TiO_2_-PU0 nanocomposite films did not show any significant differences. In all spectra, there are broad absorbance peaks in the region of 3500–3000 cm^−1^, which correspond to the stretching vibration of the hydrogen bonded, at a higher wavenumber peak [10,15], and the non-hydrogen bonded, closer to 3200 cm^−1^ [10], N–H groups [6,8,10,15]. The intense bands between 3000 and 2750 cm^−1^ correspond to the asymmetric and symmetric stretching vibrations of C–H groups [8,15], whereas the peaks around 1700 cm^−1^ are due to the stretching vibrations of the hydrogen bonded and non-hydrogen bonded carbonyl groups [39] of both urea and urethane [8,10,15]. The peaks at 1100 cm^−1^ correspond to the stretching vibration of the C–O–C ether group [8,15] of the employed PEO. The absence of a band at 2270 cm^−1^, related to NCO groups [6,8], meant that the reaction was completed and that there were not any NCO groups present in the structure of the prepared PU0 or in the nanocomposite films.

Regarding the differences between the spectra of PU0 and the nanocomposite films, the most evident one is a peak at 800 cm^−1^ corresponding to Ti–O–Ti, which widens with the increase of TiO_2_ nanoparticle content. The association of this peak is also present in the literature [8,40,41]. This widening with the increase of TiO_2_ nanoparticle content confirms the successful addition of inorganic nanoparticles to the PU0 dispersion.

### 3.2. Thermal Characterization and Stability

**Figure 3:**
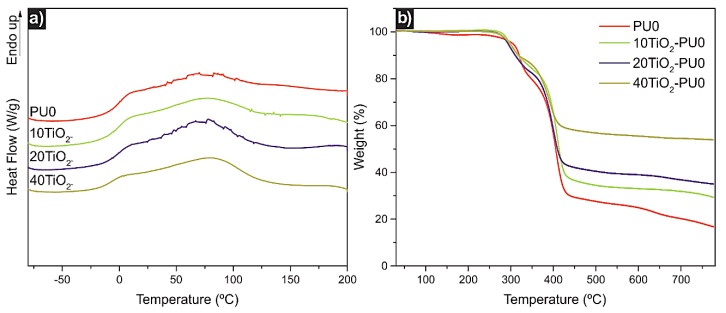
(**a**) Heating scan and (**b**) thermogravimetric analysis (TGA) curves of the PU0 and TiO_2_-PU0 nanocomposite films.

The thermal characterization of the films was performed by DSC (Figure 3a). The glass transition temperatures (*T_g_*) of all investigated films were below room temperature (Supplementary Materials Table S2). The *T_g_* of the PU0 matrix related to the hard segment decreased with the increase in TiO_2_ nanoparticle content. The *T_g_* of the PU0 matrix shifted 7 °C lower for the 40TiO_2_-PU0 nanocomposite, indicating interactions between the TiO_2_ nanoparticles and the polymer matrix. Thus, the addition of TiO_2_ nanoparticles conferred mobility to the polymer chains [7] while at the same time as acting as reinforcement [42]. This might be a consequence of the TiO_2_ nanoparticles interacting with the hard segment (MDI-SDBS segment), possibly allowing a higher soft segment (PEO) mobility.

TGA measurements were carried out in order to investigate the thermal stability of the films. Figure 3b shows the TGA curves of all the investigated materials (dTGA curves as well as TGA curve of TiO_2_ nanoparticles can be found in Supplementary Materials Figure S1 and S2). The degradation process consisted of two steps of weight loss. The first weight loss took place from 310 to 323 °C (Supplementary Materials Table S3), and corresponded to the hard segment decomposition, whereas the second weight loss occurred around 393–406 °C (Supplementary Materials Table S3), and was related to the soft segment decomposition [16]. Incorporation of TiO_2_ nanoparticles into the PU0 matrix led to a decrease in the thermal stability, as the starting decomposition temperature of the nanocomposites was lower if compared to the starting decomposition temperature of PU0. This modest decay in the thermal stability might have been the aftermath of possible interactions between the TiO_2_ nanoparticles [43], which was enhanced by the increase in TiO_2_ nanoparticle content.

The TiO_2_ nanoparticle content introduced into the PU0 matrix in each nanocomposite was determined by taking into account the difference between the residue of pristine TiO_2_ nanoparticles and the residue of each nanocomposite film.

### 3.3. Morphology

**Figure 4:**
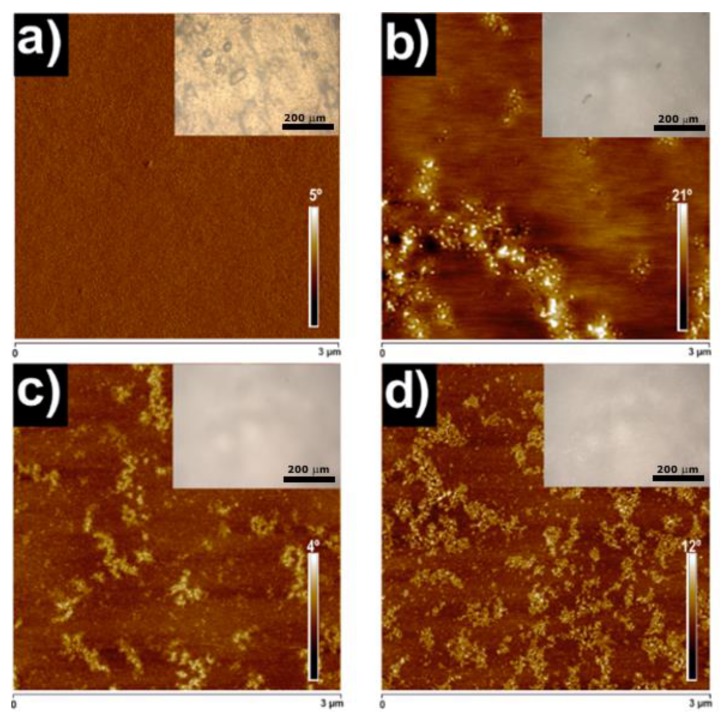
Atomic force microscopy (AFM) phase images (3 µm × 3 µm) of (**a**) PU0, (**b**) 10TiO_2_-PU0, (**c**) 20TiO_2_-PU0 and (**d**) 40TiO_2_-PU0. The inset in each AFM image corresponds to optical microscopy (OM) micrographs.

The morphology of the PU0 and nanocomposite films was studied by AFM. In addition, OM micrographs of each of the investigated films are also shown in Figure 4. As proved by the OM results, the investigated NC did not show any agglomeration of the TiO_2_ nanoparticles at the microscopic scale. As expected from our previous studies [36,44], the PU0 film did not show any phase separation (see Figure 4a and Figure S3a) between soft (PEO) and hard segments (MDI-SDBS). As a consequence, TiO_2_ nanoparticles were not located in any particular domain, since the PU0 matrix acted as a homopolymer for the investigated nanocomposites (Figure 4b–d). The TiO_2_ nanoparticles dispersed in the matrix formed spherical nanoclusters [45], given the tendency they have to interact with each other [17]. As can be clearly seen in Figure S3 of the Supplementary Materials, the size of the TiO_2_ was the same independent of the TiO_2_ nanoparticle content and cluster formation, being 25 ± 5 nm in diameter. Some of the nanoclusters were interconnected (see Figure S3c,d) [45], which could provide conductivity at a macroscopic level if they create a percolation path [46]. Nevertheless, neither OM nor AFM showed any agglomeration of the TiO_2_ nanoparticles in the polymer matrix. The amount of TiO_2_ clusters increased with the increase of TiO_2_ nanoparticle content in the TiO_2_-PU0 nanocomposites, as shown in Figure 4b–d.

### 3.4. Mechanical Properties

The mechanical properties of PU0 and TiO_2_-PU0 nanocomposite films obtained from the strain–stress curves are shown in Table 2. An increase of the TiO_2_ nanoparticle content resulted in films with higher modulus, tensile strength, and stress at break. The enhancement of the mechanical properties with an increase of TiO_2_ nanoparticles is especially visible when comparing their Young’s modulus. The Young’s modulus value of the PU0 matrix increased by 575% when 40 wt % TiO_2_ nanoparticles were added, (6.2 MPa for PU0 and 41.9 MPa for the 40TiO_2_-PU0 nanocomposite). However, the incorporation of the TiO_2_ nanoparticles led to smaller deformation at break. The deformation at break was reduced by more than half, decreasing from 490.4% for PU0 to 200.5% for 40TiO_2_-PU0. This was an expected result as the TiO_2_ nanoparticles can act as a reinforcement of the polymer matrix [9], making TiO_2_-PU0 nanocomposite films more rigid than PU0 films.

### 3.5. Self-Healing

**Figure 5:**
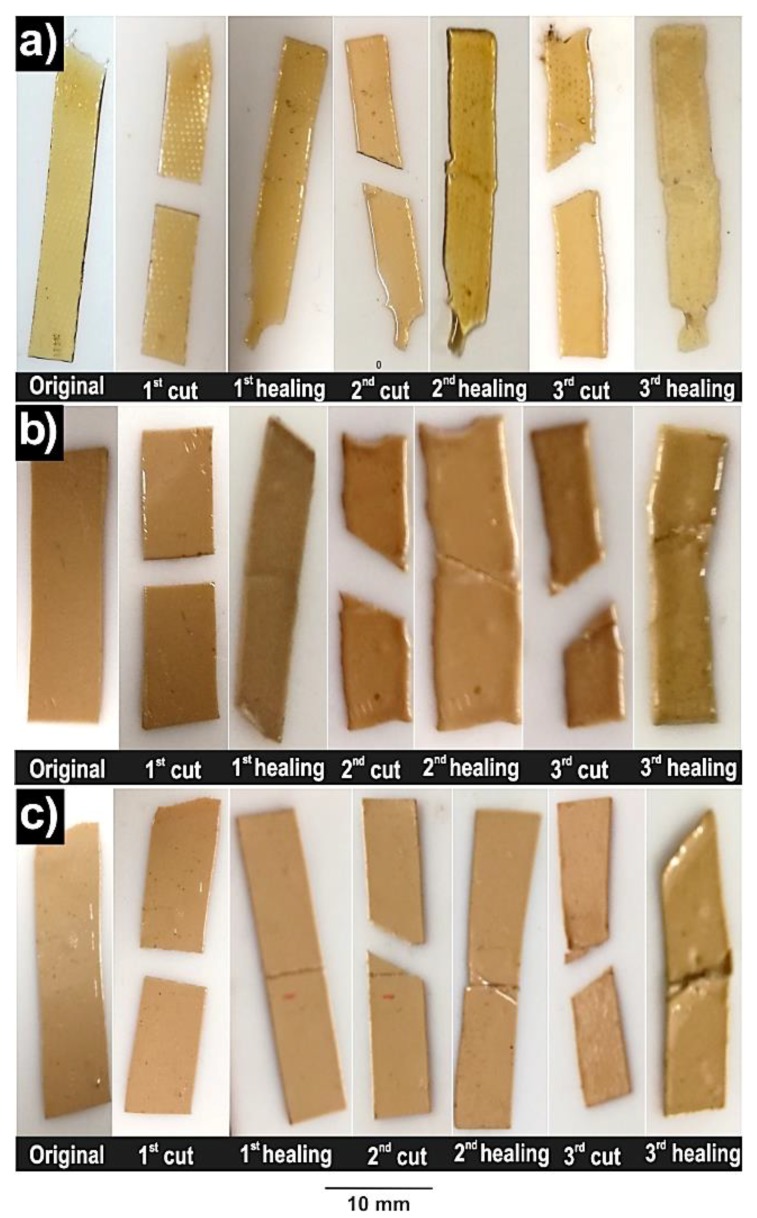
Visual self-healing process of (**a**) PU0, (**b**) 10TiO_2_-PU0 and (**c**) 20TiO_2_-PU0.

The self-healing ability of the prepared PU0 and TiO_2_-PU0 nanocomposite films was studied by means of their mechanical properties and contact angle measurements at room temperature with approximately 45% relative humidity and without any external stimuli. The 40TiO_2_-PU0 film was not analyzed since it required a considerably longer time than 24 h to recover in order to show its self-healing ability. For this measurement, nanocomposite films were cut and left to heal for three repeated cycles. Firstly, the films were cut in half and then left to heal, whereas for the second cut, the films were cut from the upper left to the lower right across the trace of the first cut and then left to heal. Finally, the third cut went from the upper right to the lower left across the trace of the first two cuts and then left to heal for the last time. The cutting/recovery cycles of each investigated film are shown in Figure 5, and the stress–strain curves are shown in the Supplementary Materials (Figure S4). The prepared materials exhibited an intrinsic self-healing ability thanks to the presence of the hydrogen bonds between the urethane and urea groups [24,26] with the ether oxygen of PEO [47], and also the hydrogen bonding of the TiO_2_ nanoparticles [3] with the poly(urethane-urea) matrix. This flexible network, together with the high urethane and urea linkages, enabled a high initial plastic deformation. Additionally, taking into account that the *T_g_* of the investigated materials was below room temperature, this conferred the investigated PU0 and TiO_2_-PU0 nanocomposite films with enough flow to ensure the recovery of the damaged area thanks to the reversible hydrogen bonding [24].

The hydrophilic nature of the prepared PU0 and TiO_2_-PU0 nanocomposite films after the successive cutting/recovery cycles was studied by static contact angle measurements. As shown in Table 3, PU0 as well as the TiO_2_-PU0 nanocomposite films showed hydrophilic characteristics. As expected, taking the hydrophilic nature of TiO_2_ nanoparticles into account, the incorporation of TiO_2_ nanoparticles into the PU0 matrix did not change the hydrophilic characteristic of the matrix [20]. The hydrophilic characteristic of the PU0 and TiO_2_-PU0 nanocomposite films decreased slightly after consecutive cutting/recovery cycles. The surface energy of both PU0 and TiO_2_-PU0 nanocomposite films (Supplementary Materials Table S4), calculated from the Young–Dupré equation using the contact angle measurements, was almost unaltered after the successive recovery cycles. The obtained value for each film and healing cycle agreed with the surface energy that can be found in the literature for polyurethane films [48].

**Figure 6:**
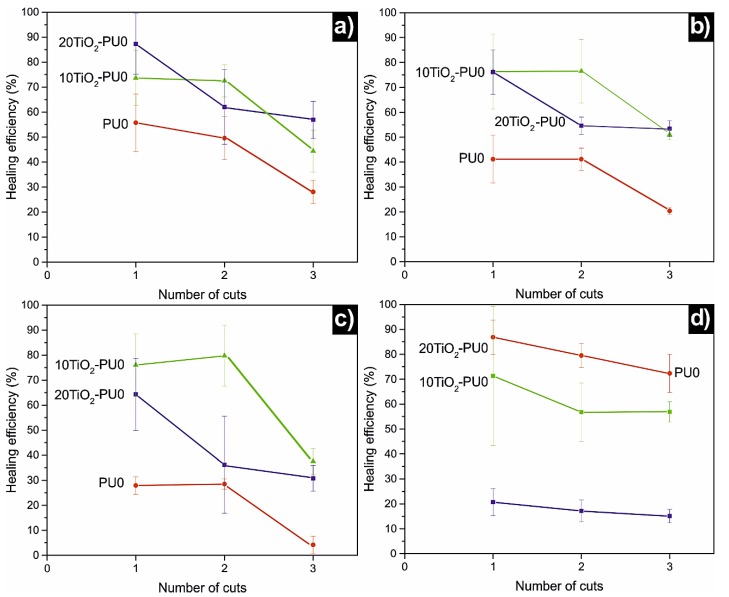
The healing efficiency of PU0, 10TiO_2_-PU0 and 20TiO_2_-PU0 for (**a**) Young’s modulus, (**b**) tensile strength, (**c**) stress at break, (**d**) deformation at break.

The healing efficiency of the self-healing ability of PU0 and TiO_2_-PU0 nanocomposite films by means of their mechanical properties is shown in Figure 6. The highest healing efficiency for tensile strength and stress at break was shown by 10TiO_2_-PU0. This tendency was quite similar also for 20TiO_2_-PU0, which showed the highest healing efficiency for Young’s modulus. Nonetheless, PU0 exhibited a high healing efficiency for deformation at break. The healing efficiency for deformation at break of 10TiO_2_-PU0 was also high in contrast with the poor healing efficiency of 20TiO_2_-PU0 for this mechanical property. Therefore, the nanocomposite films showed, in general, a greater healing efficiency than the matrix. Inasmuch as a decrease in mechanical properties after the incorporation of nanofillers is linked to inferior healing efficiency [33,49], an observed increase in mechanical properties after the incorporation of TiO_2_ nanoparticles to PU0 would have been the cause for the increase in healing efficiency that took place for the nanocomposites. This idea was supported by the fact that the healing efficiency of nanocomposites was inferior to that of PU0 due to the elongation at break and the mechanical property reduced by the incorporation of TiO_2_ nanoparticles. Nevertheless, this can be overcome by the disrupting effect over hydrogen bonding and sulfonate group interactions of the PU0 matrix that TiO_2_ nanoparticles displayed [26], resulting in a decrease in healing efficiency and leading to longer times for reparation with the increase of TiO_2_ nanoparticle content in the nanocomposites. This would explain the decrease in healing efficiency from 10TiO_2_-PU0 to 20TiO_2_-PU0, as well as the much longer time required for the 40TiO_2_-PU0 nanocomposite to self-heal.

As can be clearly observed in Figure 6, the healing efficiency decreased with the number of cuts. This could be due to the softness of the samples, which made it difficult to keep the cut specimens in direct contact during the healing process [25]. Nevertheless, the loss in the healing efficiency with successive healing cycles for intrinsic self-healing materials is a common effect [50].

### 3.6. Electrostatic Force Microscopy

EFM is a useful technique to study the electric field gradient distribution of the materials investigated above. This is a qualitative method which allows us to identify the conductive areas of the investigated materials by applying different positive and negative voltages (±V) [51,52].

**Figure 7:**
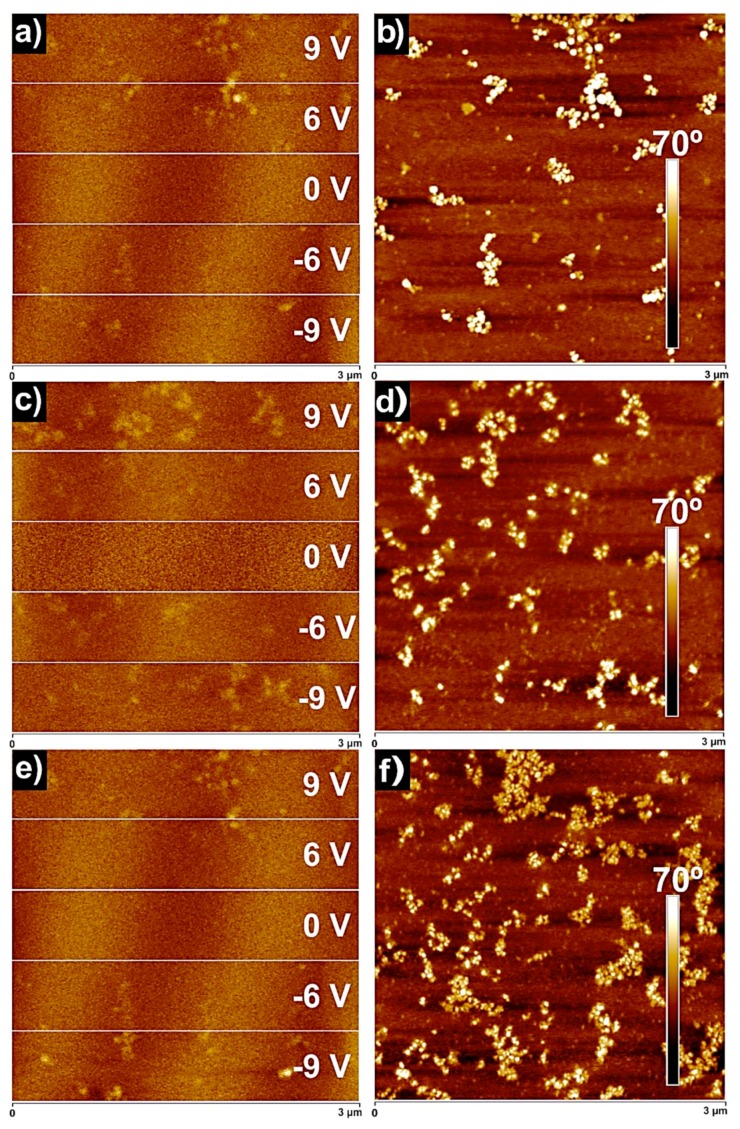
Electrostatic force microscopy (EFM) phase images (3µm × 3 µm) of (**a**) 10TiO_2_-PU0, (**c**) 20TiO_2_-PU0, (**e**) 40TiO_2_-PU0 and their simultaneously obtained AFM phase images (3 µm × 3 µm).

Firstly, in order to ensure that there was not any influence of the topography of the surface of the investigated materials on the EFM measurement, a bias of 0 V was applied. As shown in Figure 7, any charged domains were detected, confirming that the EFM measurement conditions were adequately chosen [52,53].

As expected, the locally charged TiO_2_ nanoparticles were visualized in corresponding EFM images applying a bias of 6 and 9 V. Here, it should be mentioned that in the investigated TiO_2_-PU0 nanocomposites, the only conductive components were the TiO_2_ nanoparticles [3]. An increase in the positive and negative voltage led to a higher contrast between charged and uncharged areas of the investigated nanocomposite surfaces. Consequently, the TiO_2_ nanoparticles responded in the whole range of the applied voltage, regardless of the sign of the applied V.

These findings indicate that the nanocomposite films acquired conductive behavior after the incorporation of TiO_2_ nanoparticles, as the TiO_2_ nanoparticles preserved their conductive nature. Taking into account that the weight content of the TiO_2_ nanoparticles in the prepared TiO_2_-PU0 nanocomposites was lower that the weight content corresponding to the non-conductive poly(urethane-urea), these results make TiO_2_-PU0 nanocomposite materials potential candidates for semiconductor applications [46].

## 4. Conclusions

The successful incorporation of TiO_2_ nanoparticles up to 40 wt % into synthesized waterborne poly(urethane-urea) with PEO as the soft segment was achieved by ex situ physical mixing. This successful incorporation of the titanium dioxide nanoparticles into the poly(urethane-urea) matrix was confirmed by FTIR, TGA, AFM, and EFM. An increase in the TiO_2_ nanoparticle content decreased the *T_g_* of the PU0 matrix and reduced the thermal stability. The inorganic nanoparticles formed clusters and were well-dispersed in the poly(urethane-urea) matrix, as proved by AFM. The hydrophilic nature of the PU0 did not vary significantly with the incorporation of TiO_2_ nanoparticles. As sought with the incorporation of TiO_2_ nanoparticles, Young’s modulus, and stress at break of the nanocomposite films increased as the TiO_2_ nanoparticle content increased. All investigated nanocomposite films exhibited self-healing abilities, however, the 40TiO_2_-PU0 nanocomposite required a longer period of time to heal. The healing efficiency of PU0 was the greatest for deformation at break, whereas 10TiO_2_-PU0 and 20TiO_2_-PU0 showed a greater healing ability for Young’s modulus, tensile strength, and stress at break. Nonetheless, the surface energy calculated using contact angle measurement was unaffected by the healing process. EFM corroborated that the TiO_2_ nanoparticles maintained their conductive properties after incorporation into the waterborne poly(urethane-urea). The prepared materials are interesting as a simple method for dispersing TiO_2_ nanoparticles and were employed as such, consequently leading to self-healable nanocomposite films with improved mechanical properties in which the TiO_2_ nanoparticles kept their conductive properties. These conductive nanomaterials have potential use in a broad field of applications determined by the electrical conductivity of the nanocomposites, while their self-healing ability makes them even more attractive as their lifespan is extended.

## Figures and Tables

**Table 1:** The weight average molecular weight, *M*_w_*,* polydispersity index, *PDI*, and solid content of PU0.

Sample	M_w_^a^ (g mol^−1^)	PDI ^a^	Solid content (%)
**PU0**	31,750	1.89	24.1 ± 0.6

^a^. Determined by size exclusion chromatography.

**Table 2:** Young’s modulus (*E*), tensile strength (*σ_max_*), stress at break (*σ_b_*), and deformation at break (*ε_b_*) of PU0 and TiO_2_-PU0 nanocomposite films.

Sample	*E* (MPa)	*σ_max_* (MPa)	*σ_b_* (MPa)	*ε_b_* (%)
**PU0**	6.2 ± 1.7	1.5 ± 0.2	0.7 ± 0.1	490 ± 90
**10TiO_2_-PU0**	12.6 ± 2.2	1.6 ± 0.3	1.0 ± 0.2	289 ± 20
**20TiO_2_-PU0**	25.0 ± 10.4	2.2 ± 0.3	1.3 ± 0.3	298 ± 50
**40TiO_2_-PU0**	41.9 ± 9.1	2.6 ± 0.4	2.0 ± 0.6	201 ± 73

**Table 3:** Contact angle of the original PU0 and TiO_2_-PU0 nanocomposite films and of the healed films.

Sample	Original (°)	First Healing (°)	Second Healing (°)	Third Healing (°)
**PU0**	62.4 ± 6.9	61.2 ± 8.3	53.7 ± 2.5	57.1 ± 9.5
**10TiO_2_-PU0**	65.6 ± 9.0	50.1 ± 5.5	54.9 ± 3.5	56.6 ± 4.9
**20TiO_2_-PU0**	65.7 ± 6.6	57.6 ± 2.9	57.7 ± 2.3	59.4 ± 2.9

## Supplementary Materials

The following are available online at https://www.mdpi.com/2073-4360/11/7/1209/s1, Figure S1: dTGA curves of the PU0 and TiO_2_-PU0 nanocomposite films, Figure S2: TGA curve of TiO_2_ nanoparticles, Figure S3: AFM phase images (1µm × 1 µm) of (a) PU0, (b) 10TiO_2_-PU0, (c) 20TiO_2_-PU0 and (d) 40TiO_2_-PU0, Figure S4: Stress-strain curves of original and self-healed films of (a) PU0, (b) 10TiO_2_-PU0 and (c) 20TiO_2_-PU0, Table S1: Chemical structure, molecular weight and nanoparticle concentration in water, Table S2: Glass transition temperature of the PU0 and TiO_2_-PU0 nanocomposite films, Table S3: Decomposition temperatures and TiO_2_ nanoparticles content of the investigated PU0 and TiO_2_-PU0 nanocomposites, Table S4: Surface energy of the original PU0 and TiO_2_-PU0 nanocomposite films and after each healing process.

## References

[1] Huang F., Zheng W., Tahmasbi Rad A., Nieh M.P., Cornelius C.J. (2017). SiO_2_-TiO_2_-PBC nanocomposite film morphology, solvent swelling, estimated χ parameter, and liquid transport. Polymer.

[2] Zhao L., Yu J. (2006). Controlled synthesis of highly dispersed TiO_2_ nanoparticles using SBA-15 as hard template. J. Colloid Interface Sci..

[3] Gutierrez J., Tercjak A., Mondragon I. (2010). Conductive behavior of high TiO_2_ nanoparticle content of inorganic/organic nanostructured composites. J. Am. Chem. Soc..

[4] Zhai L., Wang Y., Peng F., Xiong Z., Liu R., Yuan J., Lan Y. (2012). Synthesis of TiO_2_-SiO2/waterborne polyurethane hybrid with amino-siloxane terminated via a sol-gel process. Mater. Lett..

[5] Ma X.Y., Zhang W.D. (2009). Effects of flower-like ZnO nanowhiskers on the mechanical, thermal and antibacterial properties of waterborne polyurethane. Polym. Degrad. Stab..

[6] Zhong Z., Luo S., Yang K., Wu X., Ren T. (2017). High-performance anionic waterborne polyurethane/Ag nanocomposites with excellent antibacterial property via in situ synthesis of Ag nanoparticles. RSC Adv..

[7] Yang C.H., Liu F.J., Liu Y.P., Liao W.T. (2006). Hybrids of colloidal silica and waterborne polyurethane. J. Colloid Interface Sci..

[8] Behniafar H., Alimohammadi M., Malekshahinezhad K. (2015). Transparent and flexible films of new segmented polyurethane nanocomposites incorporated by NH_2_-functionalized TiO_2_ nanoparticles. Prog. Org. Coat..

[9] Li K., Peng J., Zhang M., Heng J., Li D., Mu C. (2015). Comparative study of the effects of anatase and rutile titanium dioxide nanoparticles on the structure and properties of waterborne polyurethane. Colloids Surfaces A Physicochem. Eng. Asp..

[10] Han J.G., Xiang Y.Q., Zhu Y. (2014). New Antibacterial composites: Waterborne polyurethane/gold nanocomposites synthesized via self-emulsifying method. J. Inorg. Organomet. Polym. Mater..

[11] Lin S.C., Ma C.C.M., Hsiao S.T., Wang Y.S., Yang C.Y., Liao W.H., Li S.M., Wang J.A., Cheng T.Y., Lin C.W. (2016). Electromagnetic interference shielding performance of waterborne polyurethane composites filled with silver nanoparticles deposited on functionalized graphene. Appl. Surf. Sci..

[12] Song W., Wang B., Fan L., Ge F., Wang C. (2019). Graphene oxide/waterborne polyurethane composites for fine pattern fabrication and ultrastrong ultraviolet protection cotton fabric via screen printing. Appl. Surf. Sci..

[13] Cakić S.M., Ristić I.S., Cincović M.M., Stojiljković D.T., Budinski-Simendić J.K. (2016). Preparation and characterization of waterborne polyurethane/silica hybrid dispersions from castor oil polyols obtained by glycolysis poly (ethylene terephthalate) waste. Int. J. Adhes. Adhes..

[14] Hendessi S., Sevinis E.B., Unal S., Cebeci F.C., Menceloglu Y.Z., Unal H. (2016). Antibacterial sustained-release coatings from halloysite nanotubes/waterborne polyurethanes. Prog. Org. Coat..

[15] Gao X., Zhu Y., Zhou S., Gao W., Wang Z., Zhou B. (2011). Preparation and characterization of well-dispersed waterborne polyurethane/CaCO_3_ nanocomposites. Colloids Surfaces A Physicochem. Eng. Asp..

[16] Zhang S., Li Y., Peng L., Li Q., Chen S., Hou K. (2013). Synthesis and characterization of novel waterborne polyurethane nanocomposites with magnetic and electrical properties. Compos. Part A.

[17] Charpentier P.A., Burgess K., Wang L., Chowdhury R.R., Lotus A.F., Moula G. (2012). Nano-TiO_2_/polyurethane composites for antibacterial and self-cleaning coatings. Nanotechnology.

[18] Yaghoubi H., Dayerizadeh A., Han S., Mulaj M., Gao W., Li X., Muschol M., Ma S., Takshi A. (2013). The effect of surfactant-free TiO_2_ surface hydroxyl groups on physicochemical, optical and self-cleaning properties of developed coatings on polycarbonate. J. Phys. D Appl. Phys..

[19] Chen X.D., Wang Z., Liao Z.F., Mai Y.L., Zhang M.Q. (2007). Roles of anatase and rutile TiO_2_ nanoparticles in photooxidation of polyurethane. Polym. Test..

[20] Abdal-hay A., Mousa H.M., Khan A., Vanegas P., Lim J.H. (2014). TiO_2_ nanorods coated onto nylon 6 nanofibers using hydrothermal treatment with improved mechanical properties. Colloids Surfaces A Physicochem. Eng. Asp..

[21] Fujishima A., Honda K. (1972). Electrochemical photolysis of water at a semiconductor electrode. Nature.

[22] Wan T., Chen D. (2017). Synthesis and properties of self-healing waterborne polyurethanes containing disulfide bonds in the main chain. J. Mater. Sci..

[23] Bekas D.G., Tsirka K., Baltzis D., Paipetis A.S. (2016). Self-healing materials: A review of advances in materials, evaluation, characterization and monitoring techniques. Compos. Part B.

[24] Garcia S.J. (2014). Effect of polymer architecture on the intrinsic self-healing character of polymers. Eur. Polym. J..

[25] Yuan C., Rong M.Z., Zhang M.Q. (2014). Self-healing polyurethane elastomer with thermally reversible alkoxyamines as crosslinkages. Polymer.

[26] Xiao Y., Huang H., Peng X. (2017). Synthesis of self-healing waterborne polyurethanes containing sulphonate groups. RSC Adv..

[27] Gao L., He J., Hu J., Wang C. (2015). Photoresponsive self-healing polymer composite with photoabsorbing hybrid microcapsules. ACS Appl. Mater. Interfaces.

[28] Zhang X., Tang Z., Tian D., Liu K., Wu W. (2017). A self-healing flexible transparent conductor made of copper nanowires and polyurethane. Mater. Res. Bull..

[29] Kim Y.J., Huh P.H., Kim B.K. (2015). Synthesis of self-healing polyurethane urea-based supramolecular materials. J. Polym. Sci. Part B Polym. Phys..

[30] Feula A., Pethybridge A., Giannakopoulos I., Tang X., Chippindale A., Siviour C.R., Buckley C.P., Hamley I.W., Hayes W. (2015). A thermoreversible supramolecular polyurethane with excellent healing ability at 45 °C. Macromolecules.

[31] Jud K., Kausch H.H., Polytechnique E. (1979). Load Transfer through chain molecules after interpenetration at interfaces. Polym. Bull..

[32] Wool R.P., O’Connor K.M. (1981). A theory of crack healing in polymers. J. Appl. Phys..

[33] Kim J.T., Kim B.K., Kim E.Y., Kwon S.H., Jeong H.M. (2013). Synthesis and properties of near IR induced self-healable polyurethane/graphene nanocomposites. Eur. Polym. J..

[34] Yamaguchi M., Ono S., Terano M. (2007). Self-repairing property of polymer network with dangling chains. Mater. Lett..

[35] Grzelak A.W., Boinard P., Liggat J.J. (2018). The influence of diol chain extender on morphology and properties of thermally-triggered UV-stable self-healing polyurethane coatings. Prog. Org. Coat..

[36] Díez-García I., Santamaría-Echart A., Eceiza A., Tercjak A. (2018). Synthesis and characterization of environmentally-friendly waterborne poly (urethane-urea) s. Eur. Polym. J..

[37] Tang H., Xu H. (2008). Process for Synthesizing 2,4-Diamino Benzene Sulfonic Acid and Its Salt. Patent WO.

[38] Schrader M.E. (1995). Young-Dupre revisted. Langmuir.

[39] Zhang S., Ren Z., He S., Zhu Y., Zhu C. (2007). FTIR spectroscopic characterization of polyurethane-urea model hard segments (PUUMHS) based on three diamine chain extenders. Spectrochim. Acta-Part A.

[40] Schrijnemakers K., Impens N.R.E.N., Vansant E.F. (1999). Deposition of a titania coating on silica by means of the chemical surface coating. Langmuir.

[41] Zhang M., Chen T., Wang Y. (2017). Insights into TiO_2_ polymorphs: Highly selective synthesis, phase transition, and their polymorph-dependent properties. RSC Adv..

[42] Zheng J., Ozisik R., Siegel R.W. (2005). Disruption of self-assembly and altered mechanical behavior in polyurethane/zinc oxide nanocomposites. Polymer.

[43] Mahfuz H., Rangari V.K., Islam M.S., Jeelani S. (2004). Fabrication, synthesis and mechanical characterization of nanoparticles infused polyurethane foams. Compos. Part A.

[44] Díez-García I., Santamaria-Echart A., Eceiza A., Tercjak A. (2018). Triblock copolymers containing hydrophilic PEO blocks as effective polyols for organic solvent-free waterborne poly (urethane-urea) s. React. Funct. Polym..

[45] Ramar A., Saraswathi R., Rajkumar M., Chen S.M. (2015). Influence of poly (n-vinylcarbazole) as a photoanode component in enhancing the performance of a dye-sensitized solar cell. J. Phys. Chem. C.

[46] Cano L., Evelyn A., Mauro D., Striccoli M., Curri M.L., Tercjak A. (2014). Optical and conductive properties of as-synthesized organic-capped TiO_2_ nanorods highly dispersible in polystyrene-block-poly (methyl methacrylate) diblock copolymer. ACS Appl. Mater. Interfaces.

[47] Mattia J., Painter P. (2007). A comparison of hydrogen bonding and order in a polyurethane and poly(urethane-urea) and their blends with poly (ethylene glycol). Macromolecules.

[48] Król P., Król B. (2012). Surface free energy of polyurethane coatings with improved hydrophobicity. Colloid Polym. Sci..

[49] Yu X., Yang P., Zhang Z., Wang L., Liu Y., Wang Y. (2018). Self-healing polyurethane nanocomposite films with recoverable surface hydrophobicity. J. Appl. Polym. Sci..

[50] Enke M., Döhler D., Bode S., Binder W.H., Hager M.D., Schubert U.S., Hager M.D., van der Zwaag S., Schubert U.S. (2016). Intrinsic self-healing polymers based on supramolecular interactions: State of the art and future directions. Self-Healing Materials.

[51] Nyffenegger R.M., Penner R.M., Schierle R. (1997). Electrostatic force microscopy of silver nanocrystals with nanometer-scale resolution. Appl. Phys. Lett..

[52] Tercjak A., Gutierrez J., Mondragon G., Mondragon I. (2011). Cellulose nanocrystals and Au nanoparticles well-dispersed in a poly (styrene-b-ethylene oxide) block copolymer matrix. J. Phys. Chem. C.

[53] Tercjak A., Garcia I., Mondragon I. (2008). Liquid crystal alignment in electro-responsive nanostructured thermosetting materials based on block copolymer dispersed liquid crystal. Nanotechnology.

