# Supplementary material for: Improvement of Mechanical Properties and Self-Healing Efficiency by Ex-Situ Incorporation of TiO2 Nanoparticles to a Waterborne Poly(Urethane-Urea)
Source: Polymers (Basel). 2019 Jul 19;11(7):1209. doi: 10.3390/polym11071209 (PMC6680434; doi:10.3390/polym11071209)
Supplement: Supplementary file 1 [file polymers-11-01209-s001.pdf]

Supplementary Materials

Improvement of mechanical properties and self-healing efficiency by *ex-situ* incorporation of TiO_2 nanoparticles to a waterborne poly(urethane-urea)

Iñigo Díez-García, Arantxa Eceiza*, Agnieszka Tercjak*

Group `Materials + Technologies ´(GMT), Department of Chemical and Environmental Engineering, Faculty of Engineering of Gipuzkoa, University of the Basque Country (UPV/EHU), Pza Europa 1, 20018 Donostia-San Sebastian, Gipuzkoa, Spain

*arantxa.aceiza@ehu.eus, agnieszka.tercjaks@ehu.eus

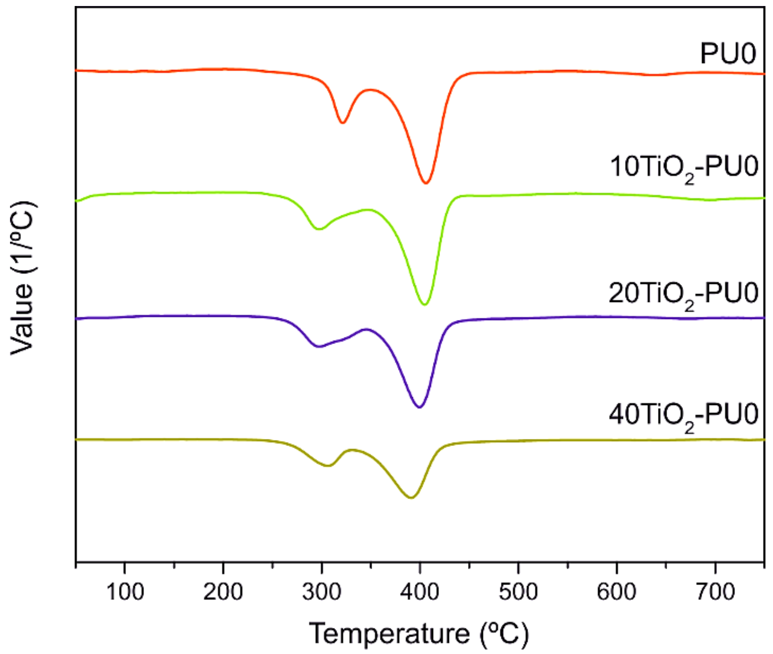

Figure S1. dTGA curves of the PU0 and TiO_2-PU0 nanocomposite films.

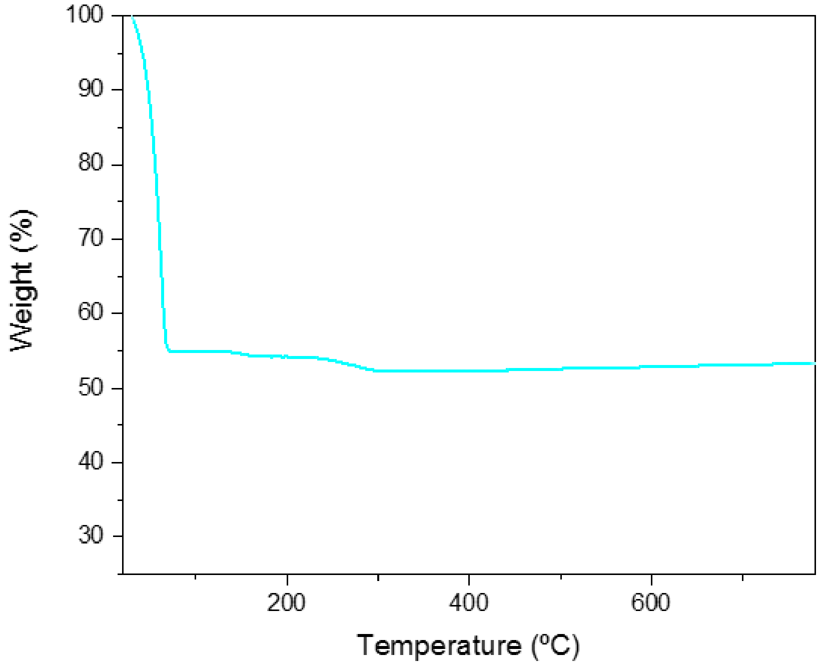

Figure S2. TGA curve of TiO_2 nanoparticles.

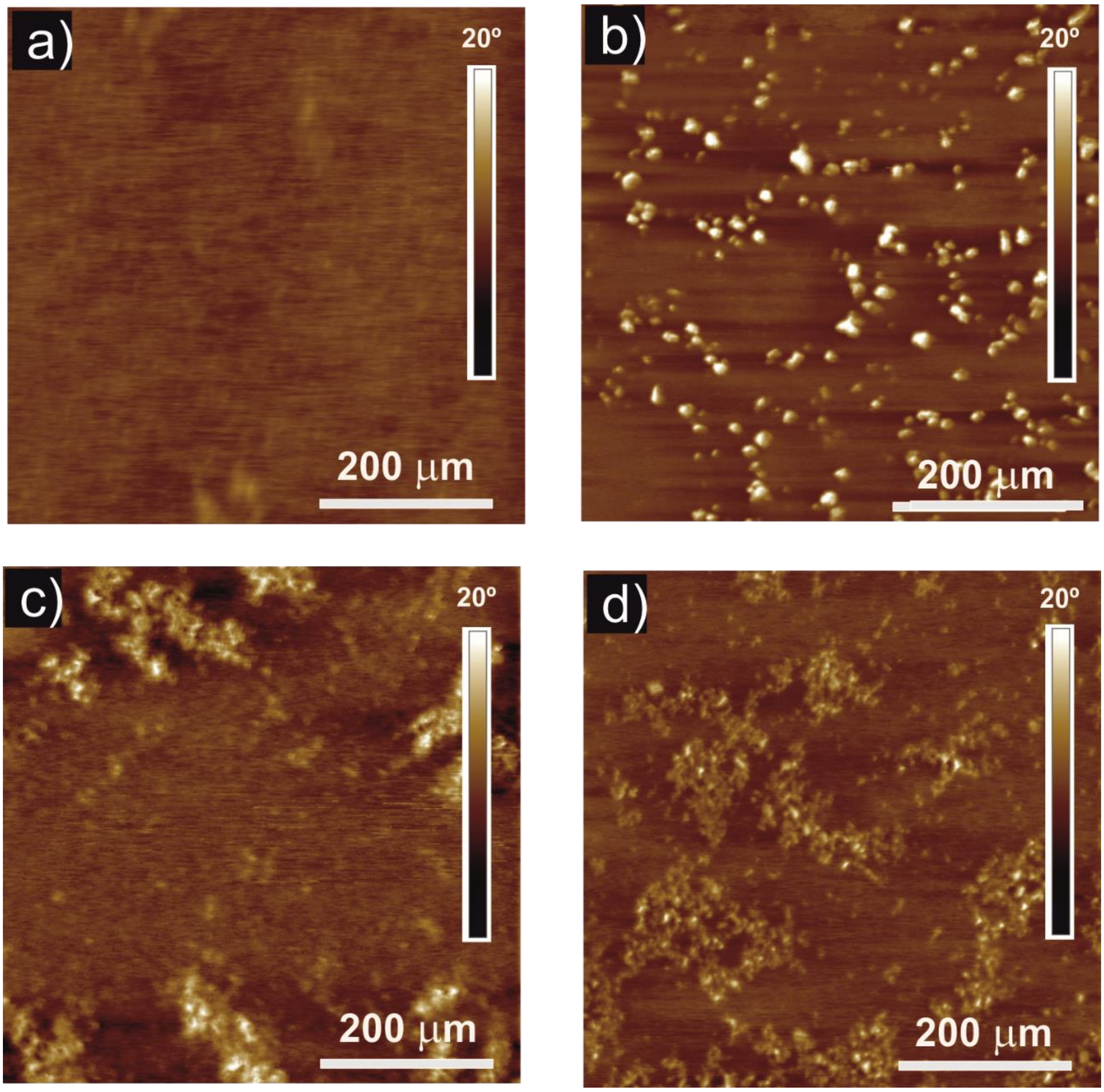

Figure S3. AFM phase images (1 μm x 1 μm) of (a) PU0, (b) $10TiO_2$-PU0, (c) $20TiO_2$-PU0 and (d) $40TiO_2$-PU0.

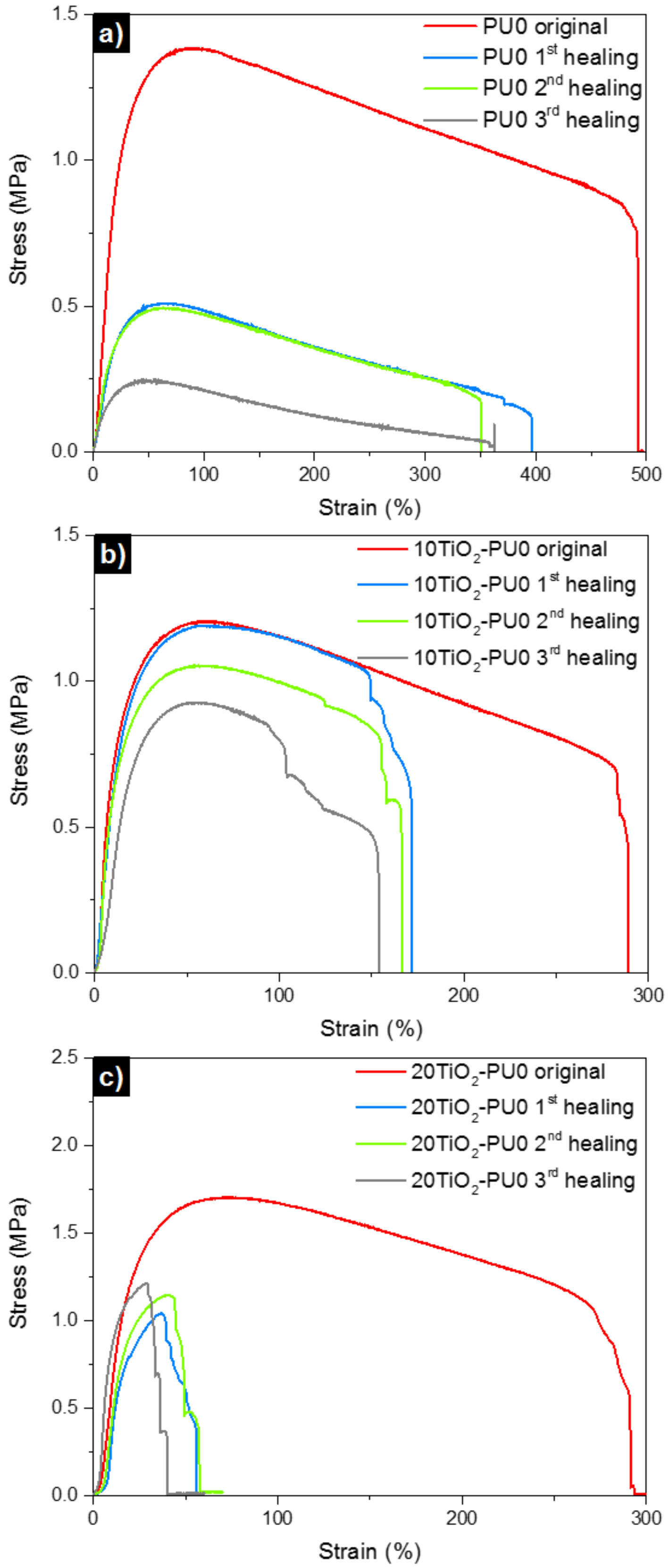

Figure S4. Stress-strain curves of original and self-healed films of (a) PU0, (b) $10TiO_2$-

PU0 and (c) 20TiO_2-PU0.

Table S1. Chemical structure, molecular weight and nanoparticle concentration in water.

Raw materials	Chemical structure	Molecular weight (g mol^{-1})	Concentration in water (wt%)
**Poly(ethylene oxide) (PEO)**	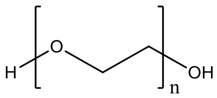	1023.00[a]	-
**2,4-Diamino-benzenesulfonic acid (DBSA)**	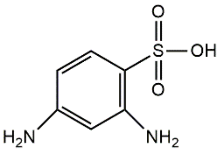	188.20	-
**4,4´-Diphenylmethane diisocyanate (MDI)**	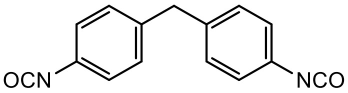	250.25	-
**Titanium dioxide nanoparticles[b] (TiO_2)**	O=Ti=O	79.86	33-37

a) *Molecular weight calculated from the hydroxyl index value determined by ASTM D4274-99*

b) *Commercial aqueous solution of TiO_2 nanoparticles*

Table S2. Glass transition temperature of the PU0 and TiO_2-PU0 nanocomposite films.

**Sample**	**T_{gSS} (℃)**
**PU0**	0
**10TiO_2-PU0**	-1
**20TiO_2-PU0**	-1
**40TiO_2-PU0**	-8

Table S3. Decomposition temperatures and TiO_2 nanoparticles content of the investigated PU0 and TiO_2-PU0 nanocomposites.

**Sample**	**T_1[a] (°C)**	**T_2[a] (°C)**	**TiO_2 wt% content (%)**
**PU0**	323	406	0
**10TiO_2-PU0**	302	403	12.6
**20TiO_2-PU0**	302	402	18.3
**40TiO_2-PU0**	311	394	37.2

a) Determined from dTGA

Table S4. Surface energy of the original PU0 and TiO2-PU0 nanocomposite films and after each healing process.

**Sample**	**Original (mN m^{-1})**	**1st healing (mN m^{-1})**	**2nd healing (mN m^{-1})**	**3rd healing (mN m^{-1})**
**PU0**	72.78 ± 0.05	72.76 ± 0.03	72.79 ± 0.03	72.80 ± 0.03
**10TiO_2-PU0**	72.79 ± 0.03	72.80 ± 0.03	72.80 ± 0.04	72.79 ± 0.03
**20TiO_2-PU0**	72.80 ± 0.03	72.78 ± 0.05	72.81 ± 0.02	72.74 ± 0.18
